# Recovery of distal coronary flow reserve in LAD and LCx after Y-Graft intervention assessed by transthoracic echocardiography

**DOI:** 10.1186/1476-7120-8-34

**Published:** 2010-08-17

**Authors:** Antonio Auriti, Vincenzo Loiaconi, Christian Pristipino, Francesco Saverio Leonardi Cattolica, Roberto Cini, Vincenzo Guido, Cinzia Cianfrocca, Salvatore Greco, Filomena Agostini, Mario Staibano, Massimo Santini

**Affiliations:** 1 Department of Cardiovascular Disease, Echocardiography Lab, S.Filippo Neri Hospital, Rome, Italy; 2 Department of Cardiovascular Disease, Heart Surgery Division, S.Filippo Neri Hospital, Rome, Italy; 3 Department of Cardiovascular Disease, Coronary Intervention Unit, S.Filippo Neri Hospital, Rome, Italy

## Abstract

**Background:**

Y- graft (Y-G) is a graft formed by the Left Internal Mammary Artery (LIMA) connected to the Left Anterior Descending Artery (LAD) and by a free Right Internal Mammary Artery (RIMA) connected to LIMA and to a Marginal artery of Left Circumflex Artery (LCx). Aim of the work was to study the flow of this graft during a six months follow-up to assess whether the graft was able to meet the request of all the left coronary circulation, and to assess whether it could be done by evaluation of coronary flow reserve (CFR).

**Methods:**

In 13 consecutive patients submitted to Y-G (13 men), CFR was measured in distal LAD and in distal LCx from 1 week after , every two months, up to six months after operation (a total of 8 tests for each patient) by means of transthoracic echocardiography (TTE) and Adenosine infusion (140 mcg/kg/min for 3-6 min). A Sequoia 256, Acuson-Siemens, was used. Contrast was used when necessary (Levovist 300 mg/ml solution at a rate of 0,5-1 ml/min). Max coronary flow diastolic velocity post-/pre-test ≥2 was considered normal CFR.

**Results:**

Coronary arteriography revealed patency of both branches of Y-G after six months. Accuracy of TTE was 100% for LAD and 85% for LCx. Feasibility was 100% for LAD and 85% for LCx. CFR improved from baseline in LAD (2.21 ± 0.5 to 2.6 ± 0.5, p = 0.03) and in LCx (1.7 ± 1 to 2.12 ± 1, p = 0.05). CFR was under normal at baseline in 30% of patients *vs *8% after six months in LAD (p = 0.027), and in 69% of patients *vs *30% after six months in LCx (p = 0.066).

**Conclusion:**

CFR in Y-G is sometimes reduced in both left territories postoperatively but it improves at six months follow-up. A follow-up can be done non-invasively by TTE and CFR evaluation.

## Background

**Figure 1:**
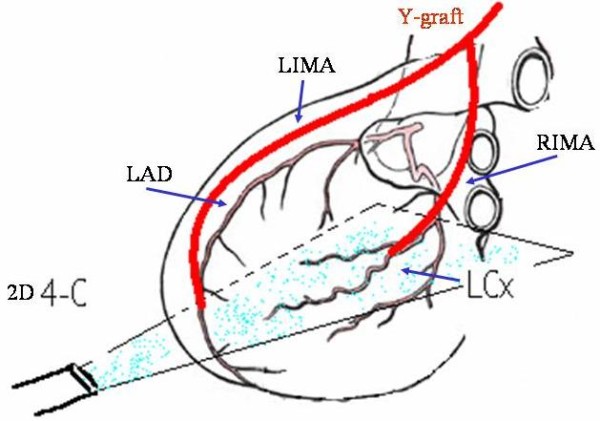
**Architecture of the Y-G with the distal LIMA and RIMA connected to the LAD an LCx; echo 4-chamber view as a reference guide to record the flow of LCx**. Abbreviations as in the text.

Y-Coronary Artery Bypass Graft (Y-G) is a surgical technique developed to limit manipulations of the ascending aorta for minimizing the possibility of embolization from aortic plaques and in desire of reaching a complete left revascularization contemporarily. The graft was formed initially by the Left Internal Mammary Artery (LIMA) and a venous graft. Now days the free Right Internal Mammary Artery (RIMA) is preferred instead of the venous graft. This kind of tailored graft with the use of a free RIMA as a bridge from LIMA and a branch of Left Circumflex Artery (LCx) has been developed after observing that arterial conduits had a better outcome in terms of patency [1]. The global graft structure is therefore formed by the LIMA connected to the Left Anterior Descending Artery (LAD) and by a free RIMA connected to the Obtuse Marginal Artery of LCx (Fig. 1). However, it has been questioned whether the LIMA was able to supply all the blood for all the left coronary circulation or if a certain reduction of the flow to the distal territories could happen under enhanced request. We hypothesized that the noninvasive study of coronary flow reserve (CFR) could assess these items[2]. Today, the noninvasive study of the graft patency and flow reserve is still usually made by recording LIMA flow from the supraclavear region or the parasternal region [3-6]. However, this way of recording may not correspond to the real conditions of distal territory and their CFR [7] for the presence of collaterals that can remain attached to the arterial conduit and because of flow competition between LAD and LCx which can lead to flow steal. The only way to gain reliable informations on the distal territories is to record the distal CFR. This can be done today by recording the flow of distal portion of recipient arteries (distal LAD and the distal LCx, in this case). The study of the distal CFR of LAD and LCx, in fact, allows to gain informations not only on the patency of the conduits but also on the status of the microcirculation of the territory supplied, on the capability of matching increased blood requests and on adaptability over time of the system after the operation. Thus, considering that there is still much to understand about, a study on the CFR in the distal territory of both LIMA and RIMA in Y-G is missed. Moreover, today, thanks to the possibility of recording the distal CFR by transthoracic echocardiography (TTE), it is possible to obtain all this informations noninvasively and in all the distal coronary territories [8-11]. In the field of this kind of artery grafts, there are studies concerning early postoperatory period [12,13], but there are very few data about a long follow-up [14,15]. Therefore, the aim of this work was to study the flow of this graft at a six months follow-up and to assess whether it could be done by noninvasive evaluation of distal CFR of LAD and LCx territories.

## Methods

### Echocardiography

**Figure 2:**
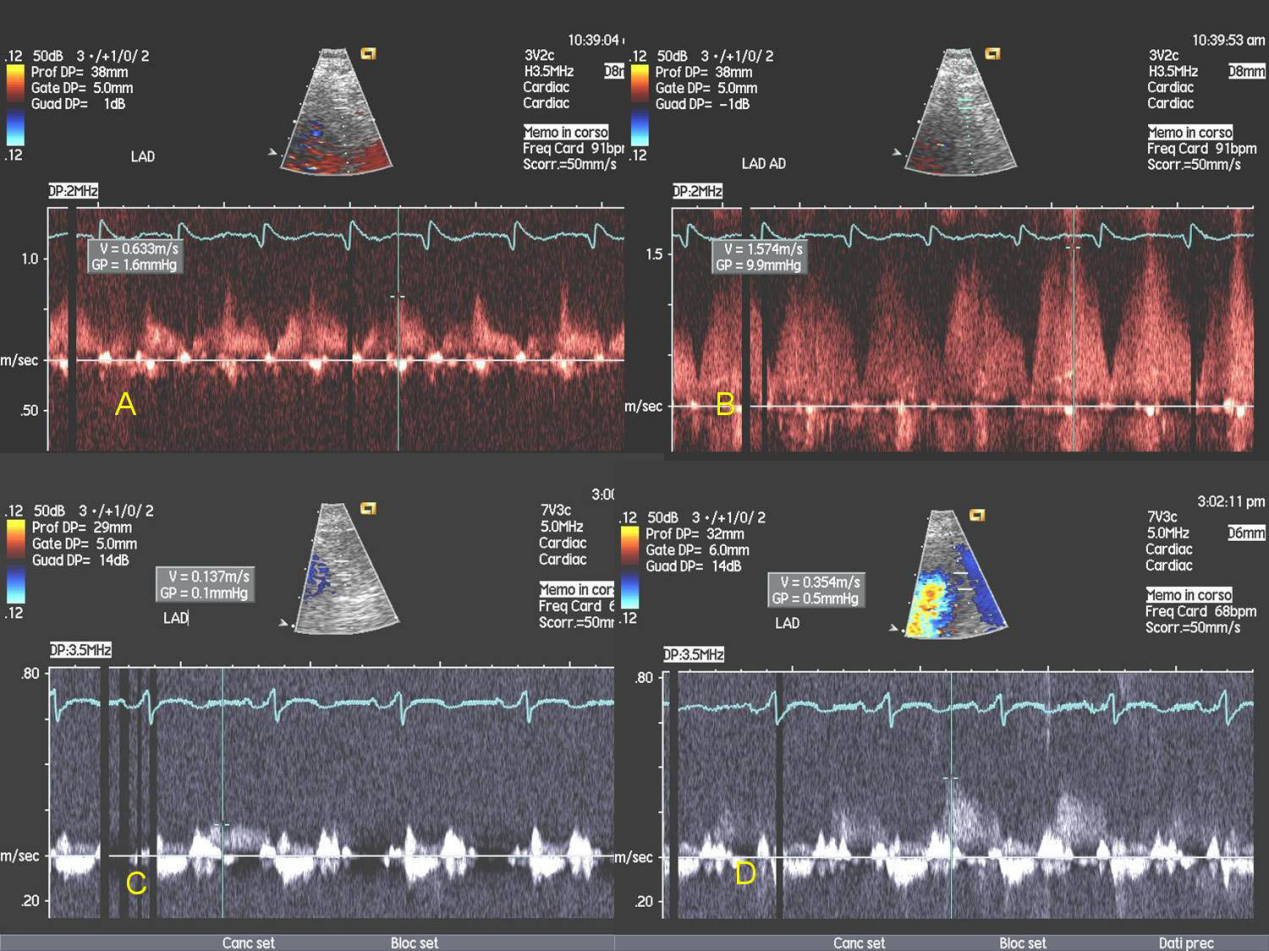
**CFR at baseline (A-B) and at six months after intervention (C-D) in LAD**. Abbreviations as in the text.

**Figure 3:**
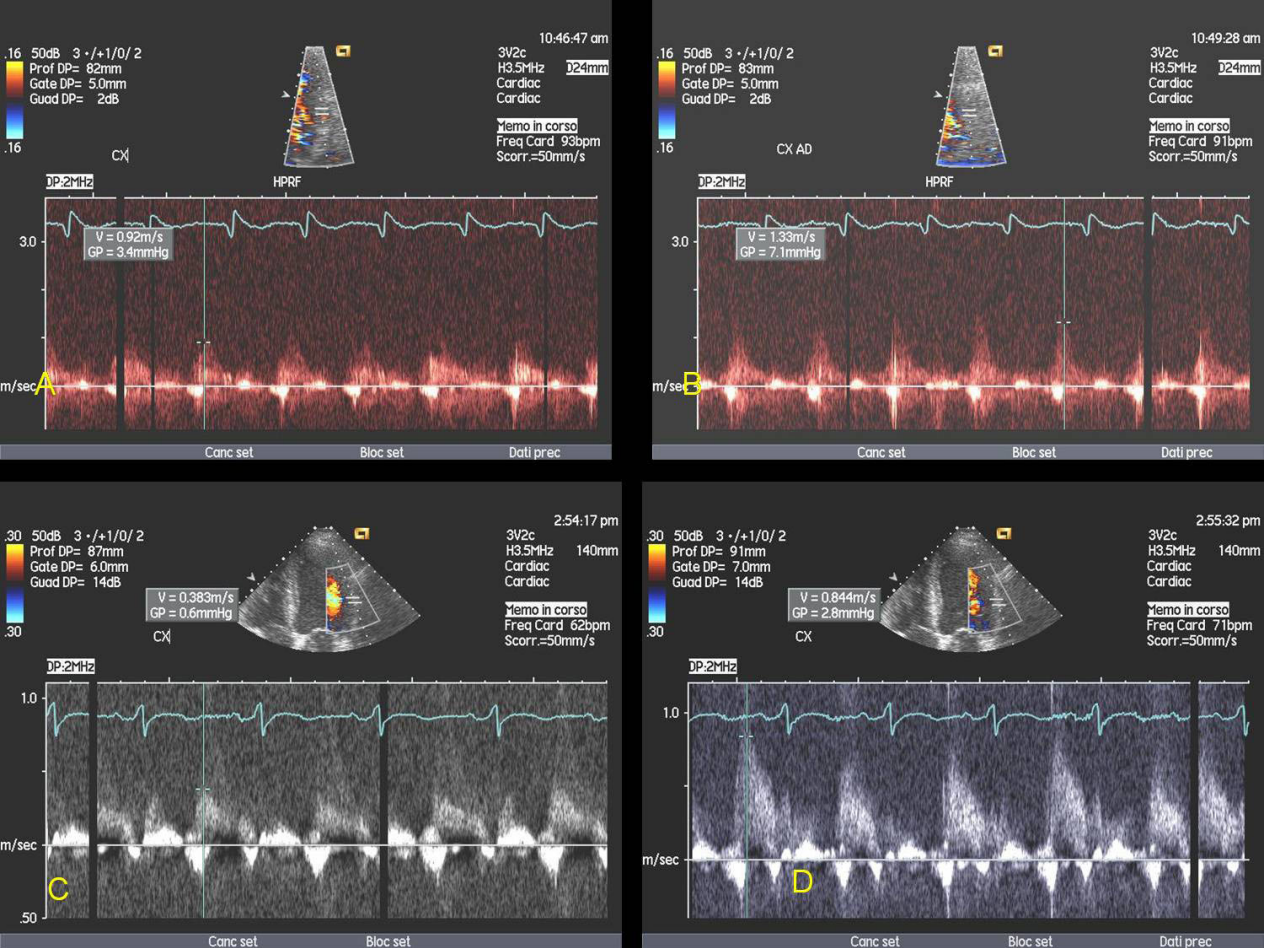
**CFR at baseline (A-B) and at six months after intervention (C-D) in LCx**. Abbreviations as in the text.

13 consecutive patients submitted to Y-G (age 62 ± 9, 13 males) 5 with prior inferior and 1 with prior anterior myocardial infarction, under standard medical therapy (including β-blockers), were enrolled after informed consent [Table 1]. CFR was calculated noninvasively by TTE in distal LAD and distal LCx by calculating the ratio of maximal coronary diastolic velocity after/to prior adenosine venous infusion (140 mcg/kg/min for 3-6 min). A 2-chamber view was used as a guide for Doppler detection of coronary flow in LAD, and a 4-chamber view was used for LCx as previously described [11,13]. An echo machine Sequoia 256-Acuson with probes of 7 MHz and 3.5 MHz was used. LAD CFR was generally recorded using a 3.5 MHz probe (and if not visualised, with a 7 MHz probe), whereas a 3.5 MHz probe was always used for LCx. Contrast agent was used when necessary (Levovist 300 mg/ml solution at a rate of 0,5-1 ml/min). A value of CFR ≥ 2 was considered normal. CFR was calculated one week after Y-G operation and, at intervals of 2-3 months, up to six months after for a total of 8 tests for each patient (Fig. 2, Fig. 3).

**Table 1:** patients' characteristics

pt	diab	hypercol	mi	hypert	LVH
1	no	yes	no	yes	no
2	no	no	no	no	no
3	yes	yes	inf	no	no
5	no	no	no	yes	no
6	yes	no	no	no	no
7	yes	no	inf	no	no
8	yes	yes	inf	yes	yes
9	yes	no	no	yes	no
10	yes	yes	inf	no	no
11	no	yes	ant	yes	no
12	no	yes	inf	yes	no
13	no	no	no	yes	no

diab: diabetes

hypercol: hypercholesterolemia

mi: prior myocardial infarction

hypert: hypertension

LVH: left ventricular hypertrophy

inf: inferior

ant: anterior

pt: patient

### Coronary arteriography

Coronary arteriography was performed with the patients' consent to assess graft patency at six months in the standard way by either femoral or radial route. Graft patency was evaluated by opacification of the conduits.

### Statistical analysis

Variables are reported as mean ± SD. χ² test for proportions comparison and paired t-test have been used. Fischer and McNemar test have been also used. A p < 0.5 has been considered significant. A software Stats and Stats has been used.

## Results

Coronary arteriography revealed patency of both branches of the Y-G in all the patients (accuracy of TTE = 100% for LAD and 85% for LCx).

**Figure 4:**
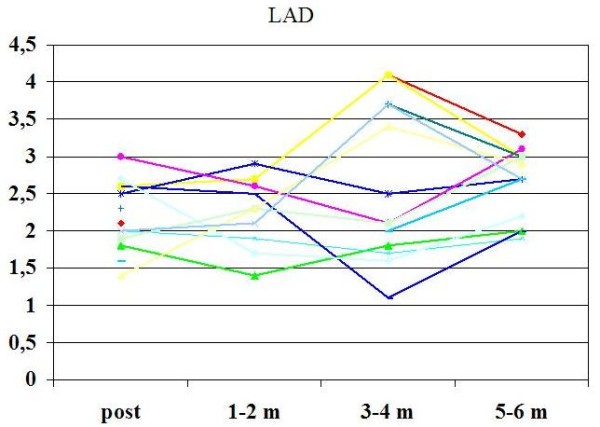
**Graphic of values of CFR in LAD during the follow-up**. Abbreviations as in the text.

**Figure 5:**
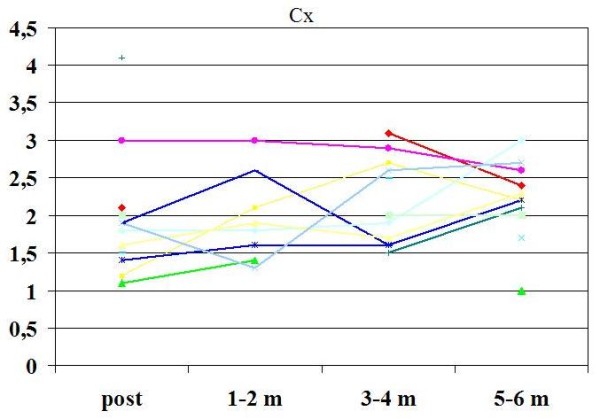
**Graphic of values of CFR in LCx during the follow up**. Abbreviations as in the text.

**Figure 6:**
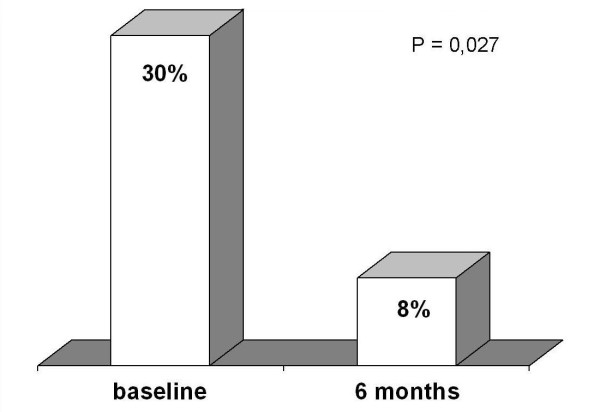
**Percent of abnormal CFR in LAD at baseline and after six months of follow-up**. Abbreviations as in the text.

**Figure 7:**
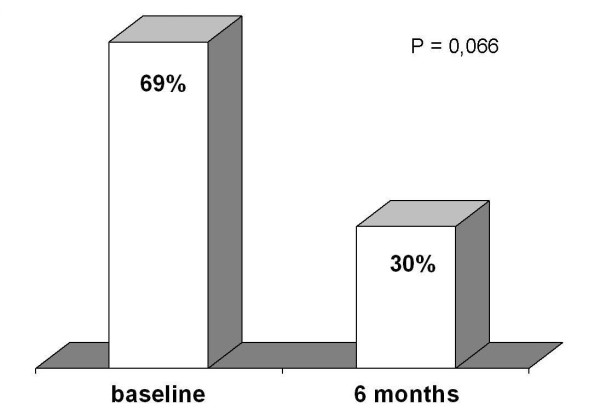
**Percent of abnormal CFR in LCx at baseline and after six months of follow-up**. Abbreviations as in the text.

Overall feasibility of CFR assessment by TTE was 100% for LAD and 85% for LCx. Contrast agent was used in 13% of cases for LAD and in 60% for LCx (p < 0.05). CFR improved from baseline to six months in LAD (2.21 ± 0.5 to 2.6 ± 0.5, p = 0.03 )(Fig 4), and in LCx (1.7 ± 1 to 2.12 ± 1, p = 0.05)(Fig. 5). Beside, CFR was under normal value in 30% of patients just after Y-G operation *vs *8% after six months in LAD (p = 0.027)(Fig 6), and in 69% of patients at baseline *vs *30% after six months in LCx (p = 0.066)(Fig. 7).

## Discussion

This study has clinical implications. One main question concerning Y-G is whether the main proximal LIMA is able to meet the requests of the whole left coronary circulation. Problems like steal phenomenon in the LCx or LAD region could appear especially in conditions of enhanced oxygen requests. In our study, it's evident a difference of CFR value between LAD and LCx territory just after Y-G operation. Although CFR is somehow impaired just after intervention in both territory, LCx territory seems to be more impaired. The reduction of CFR in LCx compared to LAD just after surgical procedure could be related to 1) reduced response of the microcirculation due to microvascular damage 2) rigidity of the graft with impaired adaptation to flow requests 3) steal mechanism from LAD territory. The latter can be hypothesized because the amount of myocardium related to LAD is greater than that of LCx with a consequent "mass effect". In our series, however, no patient displayed a negative CFR in LCx consistent with a steal phenomenon. Moreover, no patient had scarring of the lateral or posterior wall, a condition where CFR can be impaired for the reduction of microvascular bed. Beside, even if in our series some patients had conditions that can impair the CFR [Table 1], this fact shouldn't affect the results, in this case, because each patient served as a control of himself.

In our study, moreover, CFR after Y-G improved after six months both in LAD and in LCx. This could be due to 1) recovery of the microcirculation 2) adaptation of the conduit (RIMA) to the new flow 3) development of a certain wall reactivity and adaptability (although RIMA is denervated). These results are consistent with results of others Authors. In fact, Glineur et al [14] also demonstrated, invasively, a good conduit performance of LIMA and RIMA after six months. Therefore the fact that, in our series, a steal phenomenon in the region of LCx did not happen during the next months after operation but, on the contrary, CFR improved, is a reason of confidence on this kind of composite graft.

The other important result of our work, having clinical implications, is the demonstration that a good evaluation of the graft can be made noninvasively by recording the CFR of the distal recipient arteries by TTE.

However, this is a limited initial study that has to be confirmed by similar studies with longer follow-up and bigger patients' population.

### Limits

Only 13 patients could be enrolled in the study for organizative reasons unrelated to the recording of CRF, but these results could be considered preliminary and could open a field to further investigations.

## Conclusions

CFR is sometimes reduced in both branches of Y-G in several patients and especially in LCx territory postoperatively. CFR improves after six months in the LAD and LCx territories. A follow-up might then be indicated in these patients. Beside, in this work, CFR recorded by means of TTE demonstrated to be a good tool in assessing graft patency and flow dynamics noninvasively.

## List of abbreviation

Y-G: Y-graft; LAD: left anterior descending; LCx: left circumflex; TTE: transthoracic echocardiography; CFR: coronary flow reserve; RIMA: right mammary artery; LIMA: left mammary artery; 4-C: four chamber view.

## Competing interests

The authors declare that they have no competing interests.

## Authors' contributions

AA set-up the study, recorded the CFR and wrote the manuscript; VL contributed to the planning of the study, operated patients and contributed to the manuscript; CP performed coronary arteriography and contributed to the manuscript; FSLC operated the patients and contributed to the manuscript; RC operated the patients and contributed to the manuscript; VG: contributed to the manuscript; CC: contributed to the manuscript; SG: contributed to the manuscript; FA: contributed to the manuscript; MS: operated the patients and contributed to the manuscript; MSa: contributed to the manuscript.

All authors have read and approved the final manuscript.
